# Laparoscopic Splenectomy for Splenic Metastasis from Primary Lung Carcinoma

**DOI:** 10.1155/2018/2620301

**Published:** 2018-03-14

**Authors:** Carlos A. Lopera, Jean Pierre Vergnaud, Gustavo Matute-Turizo, Salin Pereira-Warr

**Affiliations:** ^1^Antioquia University, El Bosque University, SOMA Clinic, Las Vegas Clinic, El Rosario Clinic, Medellin, Colombia; ^2^School of Medicine, Pontificia Bolivariana University, LAPACI Laboratory and Integral pathology, Medellin, Colombia; ^3^Pablo Tobón Uribe Hospital, El Rosario Clinic, Medellín, Colombia

## Abstract

**Introduction:**

Isolated splenic metastases are a rare finding. Though several primary tumors can produce splenic metastases, including lung carcinoma, there are very few documented cases of isolated splenic metastases from lung carcinoma. This report presents such a case in which the splenic metastasis was removed with laparoscopic splenectomy.

**Presentation of Case:**

A 69-year-old woman with a history of lung carcinoma presented with several months of abdominal pain. Abdominal CT identified a splenic mass which was resected laparoscopically. Pathology confirmed a splenic metastasis from a primary large cell lung carcinoma.

**Discussion:**

Due to its anatomical and physiological characteristics, the spleen is a well-protected organ with respect to metastatic spread. The rarity of such metastases means that there is no evidence-based form of management. This case presents this rare metastatic occurrence and the successful management of the disease via laparoscopic splenectomy.

**Conclusions:**

This case confirms that splenic metastases can result from a primary lung carcinoma. Furthermore, the case supports successful management of this pathology by laparoscopic splenectomy.

## 1. Introduction

Splenic metastases due to lung carcinoma are exceptionally rare findings, and there are few reported cases. There is controversy within the literature as to proper management as laparoscopic splenectomy is thought to introduce the risk of peritoneal spread. This case highlights a case of large cell lung carcinoma with a splenic metastasis that was successfully managed with laparoscopic splenectomy.

## 2. Presentation of Case

The patient is a 69-year-old woman with a history of large cell carcinoma of the lung, treated with chemotherapy and radiotherapy. The primary tumor was located in the upper lobe of the right lung, measuring over 1 cm. At a follow-up, screening abdominal CT demonstrated a new contrast-enhanced mass measuring 22 mm in the spleen. Repeat CT six months later showed growth of the mass, now measuring 37 × 30 mm. Radiologically, this hypodense mass with evidence of necrosis, located in the upper pole of the spleen, was suggestive of a splenic metastasis. The patient presented with persistent diffuse abdominal pain for several months. Subsequently, she was scheduled for laparoscopic splenectomy.

Intraoperatively, a nodular mass was visualized in the upper pole of the spleen. There was no peritoneal carcinomatosis appreciated nor any other abdominal abnormalities. A traditional laparoscopic splenectomy was performed without complications. There were no postoperative complications, and the patient was discharged home two days later.

**Figure 1:**
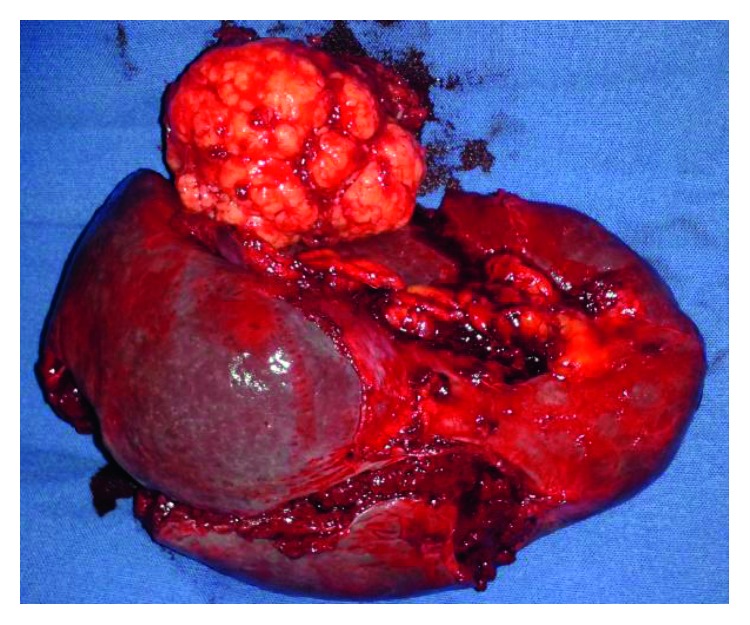
Necrotic tumor in the upper pole of the spleen.

**Figure 2:**
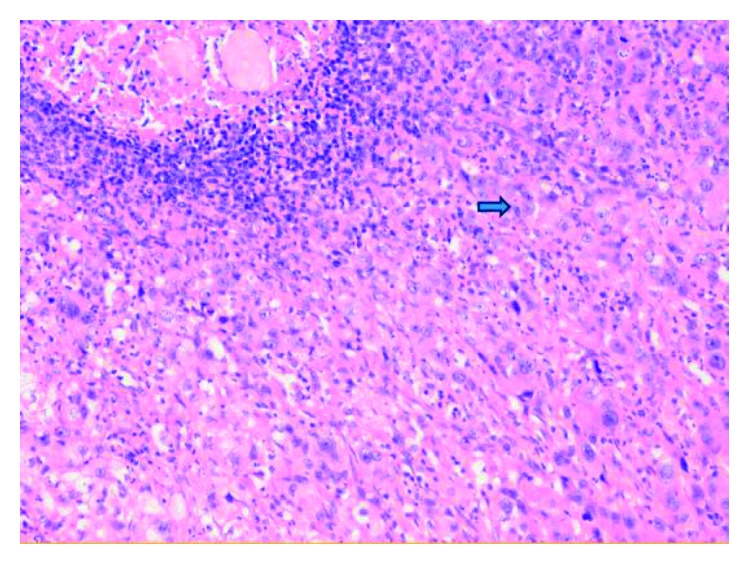
Hematoxylin and eosin staining, ×200. Microscopically, the tumor comprised epithelial cells (Arrow), with an eosinophilic cytoplasm and an abnormal core.

**Figure 3:**
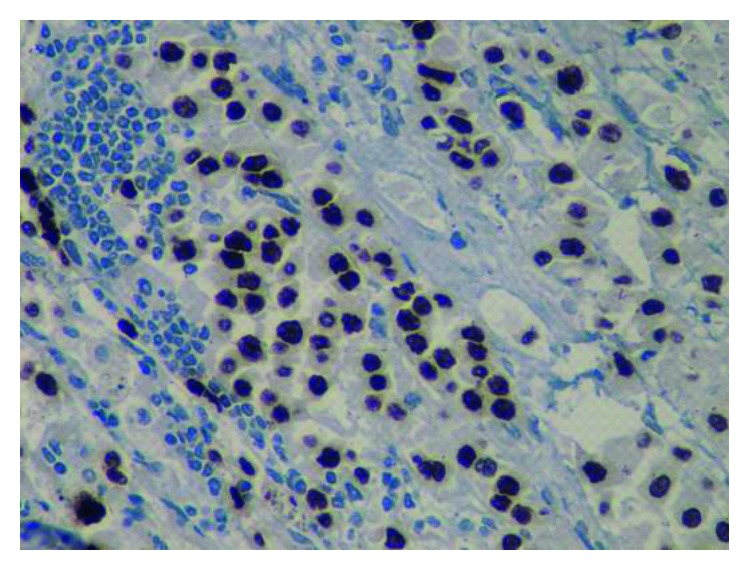
Immunostaining of the tumor was performed with TTF-1, CK7, and CK20, all of which were positive (×200).

The spleen measured 14 × 6 cm and weighed 223 grams. The necrotic tumor was 3 cm (Figure 1). Microscopically, the tumor comprised epithelial cells, with an eosinophilic cytoplasm and an abnormal core (Figure 2). Immunostaining of the tumor was performed with TTF-1, CK7, and CK20, all of which were positive (Figure 3). CK7 is a marker used to assess the reactivity of tumor membrane cells, while TTF-1 is a nuclear stain of tumor cells. Immunostaining of the tumor findings corresponds with primary neoplasm of lung carcinoma.

## 3. Discussion

It is well known that the spleen is a more protected organ from metastatic spread due to its anatomical, physiological, and lymphoid characteristics [1]. Based on autopsy studies, the prevalence of metastases from solid organ tumors ranges between 2.3 and 7.1% in the oncological population. The most common sources of metastasis are breast, lung, colorectal, and ovarian carcinomas and melanoma. The infrequency of splenic metastases is thought to be due to the high density of immune cells within the spleen, the spleen's role in “immune surveillance,” and its high concentration of the angiogenesis inhibition factor [2].

There are very few publications describing minimally invasive approaches to addressing metastatic disease in the spleen. López et al. published a case series of 6 patients with pathology-confirmed splenic metastasis from different primary neoplasms treated by laparoscopy. This form of management is believed to confer survival advantage to the patient and concurrently treat the disease [3].

Lee et al. published a larger case series with 31 patients who had splenic metastases. Of the 31 cases, 23 were of ovarian neoplasms [4].

Laparoscopic splenectomy has become the standard treatment for benign and malignant haematological disorders requiring splenectomy. The laparoscopic approach is an attractive, safe, and reliable technique in patients requiring splenectomy, as it results in a lower rate of postoperative complications including sepsis and incisional hernia and offers the patient a faster recovery [3]. However, there is a lack of evidence for the use of laparoscopic splenectomy in the management of isolated malignant tumors of the spleen, most likely due to the rarity of condition [5]. In our patient with an isolated splenic metastasis, we felt it would be the best option for management.

Although most patients with splenic metastases are clinically asymptomatic from their splenic lesions, there have been reports of painful splenomegaly, splenic vein thrombosis, and splenic rupture, making diagnosis and consideration of prompt therapeutic intervention important. Given the asymptomatic nature of these lesions, the discovery of a splenic metastasis from the initial diagnosis of a primary lung tumor can take up to 8 years [6].

Isolated splenic metastases from a primary lung cancer are extremely rare. In most reported cases of lung carcinoma with splenic metastases, the metastases are present in the context of disseminated abdominal visceral lesions.

## 4. Conclusions

Our case confirms an isolated splenic metastasis from primary large cell lung carcinoma: an extremely rare pathological finding in medical literature. In this case, laparoscopic management was the most effective and safe approach.
